# Discrimination of Fresh Tobacco Leaves with Different Maturity Levels by Near-Infrared (NIR) Spectroscopy and Deep Learning

**DOI:** 10.1155/2021/9912589

**Published:** 2021-06-07

**Authors:** Yi Chen, Jun Bin, Congming Zou, Mengjiao Ding

**Affiliations:** ^1^Yunnan Academy of Tobacco Agricultural Sciences, Kunming, China; ^2^College of Tobacco Science, Guizhou University, Guiyang, China

## Abstract

The maturity affects the yield, quality, and economic value of tobacco leaves. Leaf maturity level discrimination is an important step in manual harvesting. However, the maturity judgment of fresh tobacco leaves by grower visual evaluation is subjective, which may lead to quality loss and low prices. Therefore, an objective and reliable discriminant technique for tobacco leaf maturity level based on near-infrared (NIR) spectroscopy combined with a deep learning approach of convolutional neural networks (CNNs) is proposed in this study. To assess the performance of the proposed maturity discriminant model, four conventional multiclass classification approaches—K-nearest neighbor (KNN), backpropagation neural network (BPNN), support vector machine (SVM), and extreme learning machine (ELM)—were employed for a comparative analysis of three categories (upper, middle, and lower position) of tobacco leaves. Experimental results showed that the CNN discriminant models were able to precisely classify the maturity level of tobacco leaves for the above three data sets with accuracies of 96.18%, 95.2%, and 97.31%, respectively. Moreover, the CNN models with strong feature extraction and learning ability were superior to the KNN, BPNN, SVM, and ELM models. Thus, NIR spectroscopy combined with CNN is a promising alternative to overcome the limitations of sensory assessment for tobacco leaf maturity level recognition. The development of a maturity-distinguishing model can provide an accurate, reliable, and scientific auxiliary means for tobacco leaf harvesting.

## 1. Introduction

Harvesting plays an important role in tobacco production. The maturity largely determines the yield, quality, and economic value of tobacco leaves. Fresh tobacco leaves with optimal maturity levels have harmonious internal chemical compositions and high grade and value after flue-curing. In general, harvesting often starts two months after the transplantation of tobacco seedlings. As tobacco leaves are collected at intervals as they reach the ripe level, the maturity evaluation for tobacco leaves is manually operated [1, 2]. Accurately grasping the maturity level of tobacco leaves and timely harvesting can ensure quality production as well as better returns [3]. However, traditional maturity discrimination and harvesting of tobacco leaves based only on the appearance of tobacco leaves and experience of growers are laborious, inefficient, and quite error-prone. Thus, there is an urgent need for a reliable, rapid, and accurate automatically analyzing technique to help growers assessing the maturity levels of tobacco leaves.

In recent years, nondestructive analysis technologies have been widely used in the tobacco industry as they are fast and environment-friendly, which can significantly improve the detection speed, reduce the labor, and improve the production efficiency. Near-infrared (NIR) spectroscopy is the representative one, which can be employed to the measurements of the quality and safety attributes of tobacco and tobacco products. It has been used to determine intrinsic main chemical constituents—including total sugar, reducing sugar, nicotine, total nitrogen [4], starch, moisture, protein, K_2_O, total chlorine, heavy metals [5], ammonia, total alkaloids [6], polyphenols [7], nitrosamines, and total nitrate [8]—in tobacco leaves. In addition, numerous studies on the identification of tobacco varieties [9], tobacco parts [10], tobacco grades [11–13], aroma styles [14], and planting areas [15, 16] using NIR spectroscopy techniques have also been carried out. More specifically, the distinguishing ability of NIR spectroscopy has been evaluated to determine the maturity levels of avocados [17–20], tomatoes [21, 22], lychees [23], pomegranates [24], dates [25], table grapes [26], watermelons [27], cotton bolls [28], truffles [29], white teas [30], and peaches [31]. Despite the increasing number of applications of NIR spectroscopy in crop and fruit quality assessments, there are still only a few reports regarding the use of this technique to classify the maturity levels of fresh tobacco leaves.

Machine vision technique has been reported to rapidly evaluate the maturity levels of tobacco leaves [3, 32]. Nevertheless, the classification accuracy could be still improved. Theoretically, tobacco leaf ripening includes the mature appearance characteristics and coordination of internal chemical components [33]. The machine vision technique can be used to assess the external quality of tobacco leaves according to the color and texture features extraction, but it is challenging to correctly reflect the changes in chemical substances inside of tobacco leaves, which results in mundane recognition accuracy. In particular, it is not possible to identify a premature tobacco leaf whose appearance is very similar to that of a ripe tobacco leaf, but its internal chemical compositions do not meet the requirements of ripe tobacco leaves. NIR spectroscopy can provide more comprehensive internal and external quality information of tobacco leaves, which can be exploited for maturity classification. Hence, it is feasible to apply NIR spectroscopy to determine the quality and maturity of tobacco leaves.

Deep learning [34] is a revolutionary development of neural networks that can be used to create powerful prediction models based on multilayer abstraction to represent concepts or features. Recently, it has attracted increasing attention in various fields. As the most widely used deep learning method, convolutional neural networks (CNNs) [35, 36] with a high capability for representative feature extraction and model construction has been successfully employed to manage vibrational spectroscopic data [37–39]. Several attempts have been made, in recent years, to demonstrate the validity and feasibility. A one-dimensional convolutional neural network (1D-CNN) coupled with NIR spectroscopy has been developed to distinguish aristolochic acids analogues [40], multimanufacturers of drugs [41], waste textiles [42], peach variety [43], softwood species [44], pesticide residues on the Hami melon surface [45], the geographical origin of *T. hemsleyanum* [46], and tobacco origin [16]. The above applications achieved better discrimination results than those of shallow models.

In this study, the potential of NIR spectroscopy coupled with a deep learning method to classify the maturity levels of fresh tobacco leaves was investigated. To improve the discriminant accuracy and practical application, a 1D CNN was designed to extract more detailed features of the spectroscopic data. Specifically, the performance of the CNN classification model was assessed and compared with those of the *K*-nearest neighbor (KNN), backpropagation neural network (BPNN), support vector machine (SVM), and extreme learning machine (ELM) methods. The proposed method is a promising alternative to traditional methods for maturity level classification of tobacco leaves, which may provide an auxiliary means for objectively distinguishing the maturity levels and enhancing the quality of tobacco leaves.

## 2. Experimental Methods

### 2.1. Materials

*Nicotiana tabacum* “K326” was used in the experiment that was conducted in Dali Autonomous Prefecture, Yunnan Province, China, in 2019. The test began when the lower leaves were green and ended after the upper leaves were overmature. Since different growth positions of leaves on the same tobacco plant have obviously different internal and external quality characteristics, tobacco leaves can be divided into lower, middle, and upper leaves for harvesting. A total of 3354 representative tobacco leaf samples of the three positions were collected. The maturity of tobacco leaves was manually assessed at five levels—unripe, mature, ripe, mellow, and overmature—by several professional experts according to the rules for the curing technique of flue-cured tobacco of China (GB/T 23219-2008). The characteristics of the maturity levels of fresh tobacco leaves are shown in Table 1. Because different positions of tobacco leaves have different requirements of maturity for harvesting, the corresponding discrimination models should be established for different positions of tobacco leaves. Therefore, upper, middle, and lower tobacco leaves were separated into a training set (70%) and testing set (30%) using the Kennard–Stone method and modeled independently. Detailed sample information is presented in Table 2.

### 2.2. NIR Spectral Acquisition

**Figure 1:**
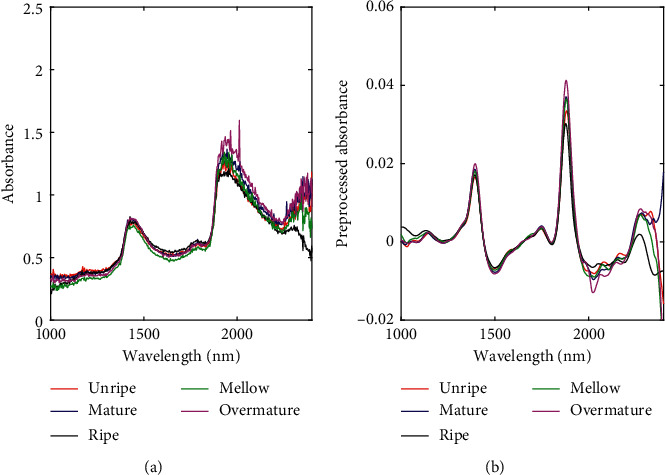
The NIR spectra of five maturity levels of upper tobacco leaves: (a) raw spectra and (b) preprocessed spectra.

All spectra of the tobacco leaves were collected by OceanView spectroscopy software in the reflectance mode using a portable extended-range near-infrared spectrometer NIRQuest256-2.5 (Ocean Optics, Inc., Dunedin, FL, USA) equipped with a linear InGaAs array detector and a standard diffuse reflection probe. The spectrometer was warmed 30 min before the sample was scanned. For each sample, six testing points were selected, avoiding leaf veins in the line of sight, evenly distributed on the tobacco leaf. The spectrum was acquired using the probe to scan tobacco leaves vertically, and the distance between them was maintained at 0.5 cm. Each spectrum was obtained through 32 scans and automatically averaged. The integration time was smaller than 200 ms. Each spectrum consisting of 512 wavelength points was obtained at intervals of 3.125 nm in the region of 900–2500 nm. The final spectrum of each tobacco leaf sample was obtained by averaging the six collected spectra.  Figure 1(a) shows an example of the collected spectra for the five maturity levels of tobacco leaves.

### 2.3. Convolutional Neural Networks (CNNs)

CNN is an efficient deep learning method proposed to minimize the preprocessing requirements of multidimensional data by sharing weights and restricting local parameters. It can autonomously learn the essential connections within the multidimensional array data through layer-by-layer feature extraction and uses four key designs to utilize the attributes of natural signals: local connection, weight sharing, pooling, and multilayer networks. As nonlinear algorithms, the CNN and BPNN have the same training method. However, the main difference is that CNN has a special structure, such as convolution and pooling, to extract and learn the internal characteristics of input data. In addition, the CNN effectively reduces the training weight and error attenuation of the network through local connection and weight sharing, so that the advantages of a multilayer neural network can be reflected.

In addition to the input, the first two stages of a typical CNN structure consist of a convolutional layer and pooling layer, which are then fully connected with the traditional multilayer perceptron (MLP), and finally the output is obtained. The elements in the convolutional layer are organized in the feature map. Each unit is connected to the local part of the upper layer through a set of weights called filters. The local weighted sum is activated by a nonlinear function. Therefore, the *k*^th^ feature graph of the convolution is defined by

(1) $$\begin{array}{c} h_{ij}^{k}\left(x\right)=f\left({\left(W^{k}\cdot x\right)}_{ij}+b_{k}\right), \end{array}$$

where **h**_*ij*_^*k*^(**x**) is the activation value of the unit in the feature map, **W**^*k*^ is the local connection weight, **b**_*k*_ is the offset value, and *f*(*z*) is the nonlinear activation function. All units in the same feature map share the same filter.

The pooling layer subsamples the local features extracted from the convolutional layer, reduces the free parameters of the network, and improves the robustness of the feature data. The pooling layer is defined by

(2) $$\begin{array}{c} x_{\text{sl}}^{k}=f\left(\beta_{l}^{k}d\left(x_{\text{cl}}^{k}\right)+b_{l}^{k}\right), \end{array}$$

where **x**_sl_^*k*^ represents the pooled output of **x**_cl_^*k*^, *d*(*z*) is the subsampled function, and *β*_*l*_^*k*^ and **b**_*l*_^*k*^ are multiplicative and additive biases, respectively.

Finally, the feature map output from the pooling layer was rasterized and fully connected to the MLP. The network parameters are estimated by solving the minimization problem of the network loss function. The weights of all filters were trained using a backpropagation algorithm.

### 2.4. Conventional Classification Techniques for Comparison

Four widely used classification algorithms—KNN [47], BPNN [48], SVM [49, 50], and ELM [51, 52]—were applied to comparatively evaluate the performance of the CNN discriminant model. The general principles of these methods are briefly described.

The KNN algorithm is a nonparametric method widely used for classification in pattern recognition. The main principle of KNN is that the category of a data point is determined according to the classification of its nearest neighbors. The algorithm operates as follows:

1. Compute the Euclidean or Mahalanobis distances from the target plot to those that were sampled
2. Sort the samples according to the calculated distances
3. Choose a heuristically optimal *k*-nearest neighbor based on the root mean square error obtained from the cross-validation
4. Calculate an inverse distance-weighted average using the *k*-nearest multivariate neighbors

BPNN, the most widely used neural network, is a type of multilayer feedforward neural network trained according to the error backpropagation algorithm. It has the abilities of arbitrary complex pattern classification and excellent multidimensional function mapping, which solves the exclusive or (XOR) and some other problems that cannot be solved by a simple perceptron. In terms of structure, the BP network has an input layer, hidden layer, and output layer. The BP algorithm uses the square of the network error as the objective function and gradient descent method to calculate the minimum value of the objective function. The calculation process of the BPNN consists of (1) a forward calculation process and (2) reverse calculation process.

SVM is a fast and reliable linear classifier based on the statistical learning theory proposed by Vapnik and Burges, which can solve high-dimensional problems, machine learning problems with small samples, and nonlinear feature interaction. The basic idea is to map the data from the original feature space to the high-dimensional feature space (Hilbert space) through a kernel function and make the linear inner product operation nonlinear. The optimal hyperplane is then established to maximize the classification interval in this space and realize the identification of unknown samples based on the hyperplane. Moreover, the SVM has strong regularization properties.

ELM is a type of single-hidden layer feedforward neural network learning algorithm according to function approximation in a finite training set, proposed by Huang and Babri. During the execution of the algorithm, the input weights of the network and bias of hidden layer neurons can be automatically adjusted, which leads to a high learning speed, good generalization performance, and unique optimal solution.

For a given training set, an excitation function, and the number of hidden layer nodes, the steps of the ELM algorithm are as follows:

1. Provide any given input weight and hidden layer bias
2. Compute the hidden layer output matrix
3. Calculate the output weight

### 2.5. Model Evaluation and Software

For actual implementation, the performance of the classification model was evaluated by calculating the discriminant accuracy (*NER*). A higher *NER* implies a higher classification capability of the model. The discriminant accuracy can be calculated by

(3) $$\begin{array}{c} \text{NER}=\frac{\sum_{g=1}^{G}n_{\text{gg}}}{n}\times 100\%, \end{array}$$

where *G* denotes the number of categories, *n* denotes the number of samples, and *n*_gg_ indicates that the samples with real class *g* are predicted to be class *g*.

All data preprocessing, KNN, BPNN, SVM, and ELM, calculations were performed using the chemometrics software Matlab 2018a (MathWorks, Inc., Natick, MA, USA). The LIBSVM (version 3.24) package was used to perform the SVM computations. In addition, the training and validation of the CNN models were implemented in Python (v3.8.2) using the Keras library (v2.4.3) and TensorFlow (v2.4.0) backend. All simulations were carried out on a laptop computer with an Intel Core 1.8 GHz CPU, 8 GB of RAM, and Windows operating system.

## 3. Results and Discussion

### 3.1. Spectral Preprocessing

Traditionally, because the NIR spectrum may contain substantial noise from the environment and instrument, preprocessing is helpful for the extraction and analysis of useful information. Different preprocessing methods lead to different prediction results. Therefore, to analyze the impacts of different pretreatment methods on the model construction, the four classical pretreatment methods first derivation, second derivation, standard normal variable transformation (SNV), and multivariate scattering correction (MSC) coupled with Savitzky–Golay smoothing and normalization were used for a comparative analysis. A total of 450 samples randomly selected from the training set of upper tobacco leaf samples were divided at a ratio of 2:1 to choose the appropriate pretreatment method. The experiment was randomly repeated five times, and the mean values were taken as the experimental results, which are shown in Table 3. Inspection of the table reveals that the discriminant accuracy after spectra processed by derivation, SNV, and MSC is improved compared with the results of the raw spectra. Relatively speaking, spectral data processed by first derivation can achieve better classification results. Thus, it was selected as the preprocessing method for the spectra of the upper, middle, and lower tobacco leaves in the subsequent classification experiments. The spectra before and after the pretreatment are shown in Figure 1. Notably, different preprocessing methods have small effects on the classification results of the CNN models. This indicates that the CNN method used to develop the NIR model is less dependent on preprocessing than other methods.

**Figure 2:**
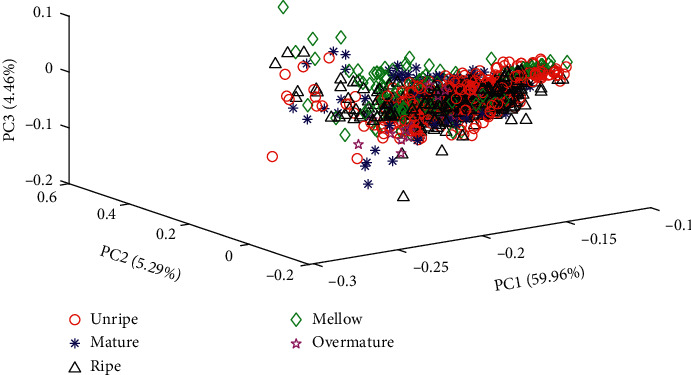
PCA score plot of the variance in NIR spectra of five maturity levels for upper tobacco leaves.

Principal component analysis (PCA) was used to cluster the spectral data of each maturity level of tobacco leaves. A PCA score plot for the five maturity levels of upper tobacco leaves is illustrated in Figure 2. It can be found that the projections of the five maturity-level samples overlap significantly and cannot be separated. In addition, the first three principal components contain only approximately 70% of the sample information. This could be explained as PCA treats all samples as a whole to find an optimal linear mapping projection with the smallest mean square error and ignores the category attribute, which may contain important separability information. Thus, it is necessary to develop a more powerful multiclassification method to discriminate different maturity levels of tobacco leaves. The CNN may be a good choice considering its strong feature extraction and learning ability.

### 3.2. CNN Discriminant Models Construction

**Figure 3:**
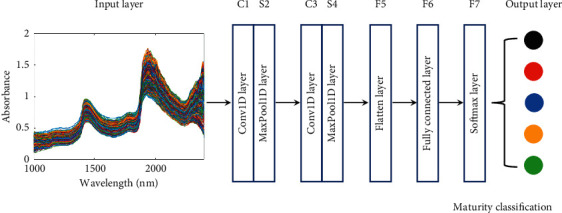
Schematic diagram of convolutional neural network model.

Based on the properties of the NIR spectra, a modified LeNet-5 CNN model was designed, which was suitable for the 1D data identification scene in this study. The basic architecture of the CNN was mainly structured into an input layer, convolutional layer, pooling layer, flatten layer, fully connected layer, and output layer. A schematic diagram of this process is shown in Figure 3. One can be observed that there are two convolutional layers. The weights of the convolutional kernel are initialized by the Xavier normal initializer. After convolution, a batch normalization mechanism is used to restandardize the activation value of the previous layer in each batch and enlarge the original reduced activation value to prevent the gradient disappearance. The pooling layer is immediately behind each convolutional layer, which can reduce the output size and risk of overfitting. The role of the global maximum pooling layer is to pool the feature map of the last layer as a whole to form a feature point, which is mainly used to solve the problem of limiting the size of the input dimension and too many parameters in the fully connected layer. The flatten layer used to flatten the multidimensional input data to 1D data is always employed as the transition from the convolutional layer to the fully connected layer. The fully connected layer is then applied to expand the feature map obtained by the last convolutional layer into a 1D vector and provide an input for the classifier. The number of neurons in the output layer is the number of maturity levels. By connecting the softmax classifier, the classification probability of the NIR data is calculated. The parameter settings of the CNN model for tobacco leaf NIR data sets are presented in Table 4.

### 3.3. Parameter Optimization for the CNN Model

To obtain a high discriminant accuracy, several key parameters should be adjusted for CNN model training. The sizes of convolutional kernel, batch size, and epoch size were investigated. 150 samples were randomly selected from the training sets of upper, middle, and lower leaf data sets as the validation sets, and the rest were used as the calibration sets for parameter adjustment, respectively. This ensured that all samples of training sets can be used for the training model. The experiment was randomly repeated five times to obtain more reliable results.

#### 3.3.1. Size of Convolutional Kernel

**Figure 4:**
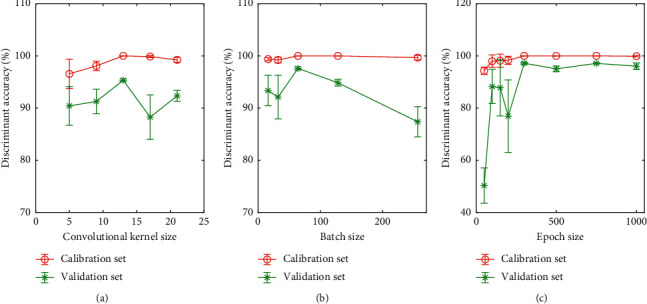
Parameters tuning of CNN model: (a) convolutional kernel size, (b) batch size, and (c) epoch size.

At first, the influence of the size of convolutional kernel on the CNN discriminant model was examined. The discriminant accuracies with the sizes of 5, 9, 13, 17, and 21 are shown in Figure 4(a), as can be seen that the size of convolutional kernel has a small effect on the CNN discriminant result. When the convolutional kernel size is set to 13, the corresponding classification accuracy of calibration and validation sets reach the maximum values. Therefore, the size of convolutional kernel was set to 13 in the CNN model construction.

#### 3.3.2. Batch Size

Since the training of the entire data set into the neural network and calculation of the gradients for a huge data set are difficult and time-consuming, batch progress is employed to divide the data set to quickly update the parameters. An appropriate batch size is helpful for a smooth model learning process. Thus, several batch sizes of 16, 32, 64, 128, and 256 were set for the experimental comparison. Discriminant results are presented in Figure 4(b). It can be seen when the batch size is 64, the highest discriminant accuracy for the validation set can be achieved. Consequently, the batch size was set to 64.

#### 3.3.3. Epoch Size

The epoch size is an important parameter in CNN model construction. If the epoch size is too small, the generalization ability of the model is not high. If the epoch size is too large, the model can easily overfit and requires a large training time. To evaluate the influence of the epoch size on the performance of the model, the discriminant results of the CNN model with epoch sizes of 50, 100, 150, 200, 300, 500, 750, and 1000 are shown in Figure 4(c). When the epoch size is small, the model is insufficiently trained with a lower classification accuracy. The classification accuracy increases with the epoch size. When the epoch size is larger than 300, the discriminant results do not significantly change and tend toward stability. Thus, the epoch size was set to 300 for the CNN modeling.

**Figure 5:**
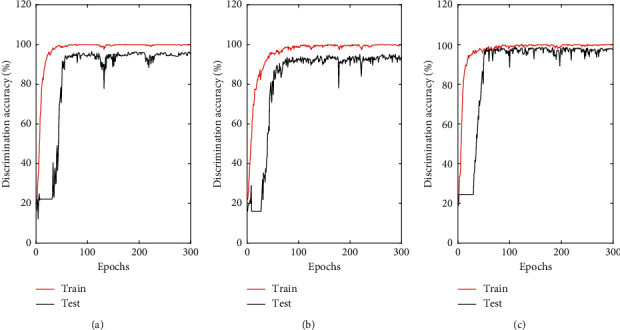
Discrimination accuracy across epochs of CNN model: (a) upper leaf, (b) middle leaf, and (c) lower leaf.

**Figure 6:**
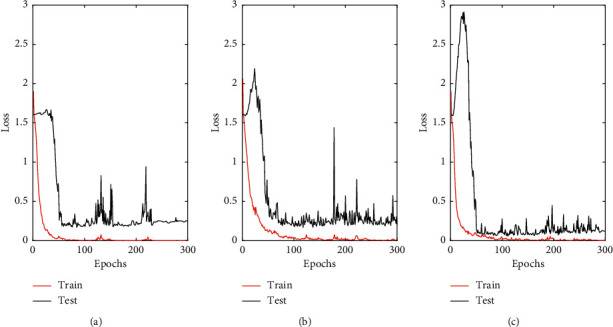
Loss function across epochs of CNN model: (a) upper leaf, (b) middle leaf, and (c) lower leaf.

The accuracy and the value of the loss function of the training set and testing set are displayed in Figures 5 and 6. As can be observed, the CNN models run stably with high accuracy. The experiment was repeated 10 times, and the mean values were taken as the final evaluation results. All experimental results are shown in Tables 5 and 6. The accuracies of the training sets of the three categories of tobacco leaf models are approximately 100%. The prediction accuracies of the three testing sets are higher than 95%. Thus, the use of the CNN method to classify and analyze NIR data sets can achieve satisfactory results. The standard deviations of the prediction results obtained by running 10 times are quite small, which indicates that the CNN models are very robust. Furthermore, CNN models can solve the discriminant problem of upper, middle, and lower tobacco leaf data sets without adjustment parameters. This suggests that the designed convolutional network has a good robustness and high generalization ability for NIR data of tobacco leaves with the help of depth networks and multiple iterations.

### 3.4. Comparative Model Analysis

To demonstrate the performance of the CNN model, KNN, BPNN, SVM, and ELM models were established for a comparative analysis in this study. A key parameter should be tuned to build a KNN classification model. A 10-fold cross-validation was used to select the appropriate number of neighbors. In the BPNN model construction, the sigmoid activation function was employed and the learning rate was set to 0.0001. The numbers of hidden layer nodes selected by BPNN running 10 times were 8, 29, 12, 24, 26, 26, 25, 21, 6, and 19 for the upper leaf data set; 28, 23, 28, 23, 8, 23, 29, 28, 24, and 19 for the middle leaf data set; and 23, 25, 3, 22, 20, 1, 1, 21, 26, and 21 for the lower leaf data set. To establish the SVM model, the radial basis function (RBF) was used as the kernel function, while the sigmoid function was selected as the excitation function. Furthermore, a grid search algorithm was used to optimize the penalty parameter and kernel function parameter. In addition, the numbers of hidden layer nodes selected by ELM running 10 times were 196, 179, 194, 131, 193, 166, 129, 124, 151, and 148 for the upper leaf data set; 92, 153, 124, 162, 181, 124, 148, 195, 126, and 154 for the middle leaf data set; and 121, 73, 171, 117, 93, 98, 194, 185, 186, and 142 for the lower leaf data set. All optimal parameters of these four models are listed in Table 7.

The classification accuracies of the three tobacco leaf data sets predicted by the KNN, BPNN, SVM, and ELM methods are listed in Table 6. The CNN models outperform the other methods in terms of the maturity level judgment of tobacco leaves. The prediction accuracies of the CNN models for the upper, middle, and lower tobacco leaf data sets are increased by 14.47%, 12.11%, and 8.4% compared with those of the KNN models, respectively. Compared with those of the BPNN models, the classification accuracies of the CNN models are jumped by 44.87%, 18.73%, and 12.1%, respectively. The prediction accuracies are largely improved, which reflects the powerful feature extraction and learning ability of the CNN model. Compared with those of the SVM models, the classification accuracies of the CNN models for the upper, middle, and lower tobacco leaf data sets are up by 4.86%, 6.69%, and 4%, respectively. In addition, SVM models achieve better prediction accuracies than those of the other three methods, possibly as the SVM maps input vectors to the feature space and builds a hyperplane to accomplish classification using a kernel function. What is more, the prediction results of the CNN models are better than those of the ELM models with the classification accuracies for the upper, middle, and lower leaf data sets improved by 9.83%, 9.79%, and 5.19%, respectively. Overall, the analysis and comparison confirm the excellent classification ability of the CNN model to discriminate the maturity levels of tobacco leaves. This reveals that the superiority of deep learning models with a high ability for feature extraction and learning over shallow learning models.

## 4. Conclusions

In this study, the potential of NIR spectroscopy coupled with a deep learning method to classify the maturity levels of fresh tobacco leaves was investigated. NIR spectroscopy is a useful tool to determine the internal and external qualities of tobacco leaves precisely and nondestructively. A simple 1D CNN-based classification method with two convolutional layer structures was designed to establish a discriminant model for the spectroscopic data of fresh tobacco leaves. Results of experimental analysis indicated that the CNN models yielded high discriminant accuracies of 96.18%, 95.2%, and 97.31% for the upper, middle, and lower leaf data sets, respectively, superior to those of the KNN, BPNN, SVM, and ELM models. The CNN method, which has a strong feature extraction and learning ability, has a beneficial effect on the classification accuracy. Thus, CNN is a promising alternative to traditional methods for maturity level classification of tobacco leaves based on NIR spectroscopy. The developed technique can provide discriminant results without sample preparation procedures, which can significantly help growers in terms of decisions regarding the proper harvest time in the field. Further studies should be carried out before the application on tobacco leaves harvested from a complex agricultural environment.

## Figures and Tables

**Table 1:** The characteristics of fresh tobacco leaves in five maturity levels.

Maturity levels	Characteristics description of fresh tobacco leaf
Unripe	Leaf color is dark green without any yellow, the main vein and branches are all green, and pubescence is not fallen off.
Mature	Leaf color is light green with litter yellow, about 2/3 main vein turns white, and the branches are green with a small amount of pubescence shedding.
Ripe	Leaf color is yellow-green, the main vein is all white, about 1/3 branches turn white, pubescence partly falls off, and the leaf tip is slightly hung down.
Mellow	Leaf color is yellow, the main vein is all white and bright, about 2/3 branches turn white, pubescence is basically or mostly shed off, the leaf surface is covered with macula, the leaf tip and leaf edge turn white, slightly withered, and the leaf tip is scorched and hooked down.
Overmature	The main vein and branches are all white and bright, and leaf color is yellow-white. Most of pubescence fall off, the leaf ear is yellow with withered sharp and scorched edge.

**Table 2:** The detail of tobacco leaves data sets.

Data sets	Total samples	Training set	Testing set	Unripe	Mature	Ripe	Mellow	Overmature
Upper leaves	1128	790	338	219	225	226	229	229
Middle leaves	1085	760	325	216	222	218	219	210
Lower leaves	1141	799	342	232	227	235	228	219

**Table 3:** Discriminant accuracy (%) of different preprocessing methods.

Preprocessing methods	KNN	BPNN	SVM	ELM	CNN
Raw	55.33	77.05 ± 3.61	87.33	72.4 ± 3.25	92.35 ± 2.61
First derivation	85.33	88.32 ± 2.69	93.33	82.46 ± 4.44	95.84 ± 1.25
Second derivation	84.67	85.1 ± 2.54	92.67	80.24 ± 5.91	94.55 ± 1.65
SNV	74	86.67 ± 2.91	94	85.03 ± 3.43	94.36 ± 1.24
MSC	74	86.5 ± 2.52	93.33	84.49 ± 1.66	93.38 ± 1.42

**Table 4:** Parameter settings of the convolutional neural networks for upper, middle, and lower leaves data sets.

Layers	Model parameters	Output shape
Input layer	NIRS data of 454 × 1 dimension	
Conv1D (*C*1)	128 convolutional kernels of the size 13 × 1, the Relu function and BN mechanism, stride = 1	450 × 128
MaxPooling1D (*S*2)	Maxpooling, pooling size = 2 × 1, stride = 1	225 × 128
Conv1D (*C*3)	64 convolutional kernels of the size 13 × 1, the Relu function and BN mechanism, stride = 1	221 × 64
MaxPooling1D (*S*4)	Maxpooling, pooling size = 1 × 1, stride = 1	221 × 64
Flatten (*F*5)	Flatten the feature vector of the *S*4 layer into 1 vector	14144 × 1
Dense (*F*6)	100 output neurons fully connected to all neurons in layer *F*5, the Relu function	100 × 1
Dense (*F*7)	5 output neurons consistent with the number of maturity levels	5 × 1
Output layer	The softmax function	

**Table 5:** The prediction results (%) of convolutional neural networks running 10 times.

Data sets	Sample sets	Discriminant accuracy
*Upper leaves*	Training set	99.75, 99.87, 99.75, 99.75, 100, 100, 100, 100, 100, 100
	Testing set	95.86, 95.86, 96.15, 96.45, 95.86, 96.15, 96.45, 96.15, 96.45, 96.45

*Middle leaves*	Training set	100, 99.87, 100, 99.74, 99.61, 99.08, 98.95, 99.34, 99.61, 99.34
	Testing set	95.38, 94.77, 94.46, 94.77, 95.69, 95.69, 95.38, 95.69, 95.08, 95.08

*Lower leaves*	Training set	99.12, 99.75, 99.75, 99.62, 100, 99.75, 99.75, 99, 99.5, 99.75
	Testing set	96.49, 97.08, 97.37, 98.54, 97.66, 97.95, 96.2, 96.49, 97.37, 97.95

**Table 6:** The prediction results (%) of convolutional neural networks and other four methods.

Data sets	Sample sets	KNN	BPNN	SVM	ELM	CNN
*Upper leaves*	Training set	89.87	92.11 ± 0.46	96.2	96.11 ± 1.82	99.91 ± 0.12
	Testing set	84.02	66.39 ± 7.31	91.72	87.57 ± 2.79	96.18 ± 0.26

*Middle leaves*	Training set	90.79	93.66 ± 0.79	93.03	94.24 ± 2.1	99.55 ± 0.37
	Testing set	84.92	80.18 ± 2.93	89.23	86.71 ± 1.16	95.2 ± 0.44

*Lower leaves*	Training set	91.99	95.87 ± 0.43	94.87	95.71 ± 2.28	99.6 ± 0.31
	Testing set	89.77	86.81 ± 4.06	93.57	92.51 ± 2.12	97.31 ± 0.75

**Table 7:** The optimal parameters for KNN, BPNN, SVM, and ELM.

Data sets	Parameters				
	KNN	BPNN	SVM		ELM
	*Number of neighbors*	*Number of hidden layer nodes*	*Penalty parameter*	*Kernel function parameter*	*Number of hidden layer nodes*
Upper leaves	6	19.6 ± 8.15	32	0.0313	161.1 ± 28.38
Middle leaves	5	23.3 ± 6.25	8	0.0625	145.9 ± 30.34
Lower leaves	8	16.3 ± 10.27	16	0.0313	138 ± 43.89

## Data Availability

The spectral data used to support the findings of this study are currently under embargo, while the research findings are commercialized. Access to data is restricted because of commercial confidentiality. Requests for data, 12 months after publication of this article, will be considered by the corresponding author.
